# Field-Evaluation of a New Lateral Flow Assay for Detection of Cellular and Humoral Immunity against *Mycobacterium leprae*

**DOI:** 10.1371/journal.pntd.0002845

**Published:** 2014-05-08

**Authors:** Kidist Bobosha, Elisa M. Tjon Kon Fat, Susan J. F. van den Eeden, Yonas Bekele, Jolien J. van der Ploeg-van Schip, Claudia J. de Dood, Karin Dijkman, Kees L. M. C. Franken, Louis Wilson, Abraham Aseffa, John S. Spencer, Tom H. M. Ottenhoff, Paul L. A. M. Corstjens, Annemieke Geluk

**Affiliations:** 1 Department of Infectious Diseases, Leiden University Medical Center, Leiden, The Netherlands; 2 Armauer Hansen Research Institute, Addis Ababa, Ethiopia; 3 Department of Molecular Cell Biology, Leiden University Medical Center, Leiden, The Netherlands; 4 Department of Microbiology, Immunology & Pathology, Colorado State University, Fort Collins, Colorado, United States of America; University of Tennessee, United States of America

## Abstract

**Background:**

Field-applicable tests detecting asymptomatic *Mycobacterium leprae* (*M. leprae*) infection or predicting progression to leprosy, are urgently required. Since the outcome of *M. leprae* infection is determined by cellular- and humoral immunity, we aim to develop diagnostic tests detecting pro-/anti-inflammatory and regulatory cytokines as well as antibodies against *M. leprae*. Previously, we developed lateral flow assays (LFA) for detection of cytokines and anti-PGL-I antibodies. Here we evaluate progress of newly developed LFAs for applications in resource-poor settings.

**Methods:**

The combined diagnostic value of IP-10, IL-10 and anti-PGL-I antibodies was tested using *M. leprae*-stimulated blood of leprosy patients and endemic controls (EC). For reduction of the overall test-to-result time the minimal whole blood assay time required to detect distinctive responses was investigated. To accommodate LFAs for field settings, dry-format LFAs for IP-10 and anti-PGL-I antibodies were developed allowing storage and shipment at ambient temperatures. Additionally, a multiplex LFA-format was applied for simultaneous detection of anti-PGL-I antibodies and IP-10. For improved sensitivity and quantitation upconverting phosphor (UCP) reporter technology was applied in all LFAs.

**Results:**

Single and multiplex UCP-LFAs correlated well with ELISAs. The performance of dry reagent assays and portable, lightweight UCP-LF strip readers indicated excellent field-robustness. Notably, detection of IP-10 levels in stimulated samples allowed a reduction of the whole blood assay time from 24 h to 6 h. Moreover, IP-10/IL-10 ratios in unstimulated plasma differed significantly between patients and EC, indicating the feasibility to identify *M. leprae* infection in endemic areas.

**Conclusions:**

Dry-format UCP-LFAs are low-tech, robust assays allowing detection of relevant cytokines and antibodies in response to *M. leprae* in the field. The high levels of IP-10 and the required shorter whole blood assay time, render this cytokine useful to discriminate between leprosy patients and EC.

## Author Summary

Leprosy is one of the six diseases considered by WHO as a major threat in developing countries and often results in severe, life-long disabilities and deformities due to delayed diagnosis. Early detection of *Mycobacterium leprae* (*M. leprae*) infection, followed by effective interventions, is considered vital to interrupt transmission. Thus, field-friendly tests that detect asymptomatic *M. leprae* infection are urgently required. The clinical outcome after *M. leprae* infection is determined by the balance of pro- and anti-inflammatory cytokines and antibodies in response to *M. leprae*. In this study, we developed lateral flow assays (LFA) for detection of pro-inflammatory (IP-10) vs. anti-inflammatory/regulatory (IL-10) cellular immunity as well as antibodies against *M. leprae* and evaluated these in a field setting in Ethiopia using lightweight, portable readers. We show that detection of IP-10 allowed a significant reduction of the overall test-to-result time from 24 h to 6 h. Moreover, IP-10/IL-10 ratios in unstimulated plasma differed significantly between patients and EC, which can provide means to identify *M. leprae* infection. Thus, the LFAs are low-tech, robust assays that can be applied in resource-poor settings measuring immunity to *M. leprae* and can be used as tools for early diagnosis of leprosy leading to timely treatment and reduced transmission.

## Introduction

Leprosy, a curable infectious disease caused by *Mycobacterium leprae* (*M. leprae*) that affects the skin and peripheral nerves, is one of the six diseases considered by the WHO as a major threat in developing countries [1]. Despite being treatable, leprosy often results in severe, life-long disabilities and deformities [2] due to delayed- or misdiagnosis. Transmission of leprosy is clearly unabated as evidenced by the number of new cases, 10% of whom are children, that plateaued at nearly 250,000 each year since 2005 [1]. Continued transmission in endemic areas likely occurs from the large reservoir of individuals who are infected subclinically. Thus, early detection of *M. leprae* infection, followed by effective interventions, is considered vital to interrupt transmission as highlighted by the WHO 2011–2015 global strategy [3]. Despite this pressing need, field-friendly tests that detect asymptomatic *M. leprae* infection are lacking, nor are there any biomarkers known that predict progression to disease in infected individuals.

Lateral flow assays (LFAs), are simple immunochromatographic assays detecting the presence of target analytes in samples without the need for specialized and costly equipment. Combinations of LFAs with up-converting phosphor (UCP) reporter technology are useful for detection of a variety of analytes, e.g., drugs of abuse [4], protein and polysaccharide antigens from pathogens like *Schistosoma* and *Brucella* [5], [6], bacterial and viral nucleic acids [7], [8] and antibodies against *M. tuberculosis*, HIV, hepatitis virus and *Yersinia pestis* [9]–[11]. The phosphorescent reporter utilized in UCP-LFAs is excited with infrared light to generate visible light, a process called up-conversion. UCP-based assays are highly sensitive since up-conversion does not occur in nature, avoiding interference by autofluorescence of other assay components. Importantly, UCP-LF test strips can be stored as permanent records allowing re-analysis in a reference laboratory.

In leprosy, the innate and adaptive immune response to *M. leprae* matches the clinical manifestations as substantiated by the characteristic spectrum ranging from strong Th1 immunity in tuberculoid leprosy to high antibody titers to *M. leprae* with Th2 cytokine responses in lepromatous leprosy [12]. In view of this spectral character, field-applicable tests for leprosy should allow simultaneously detection of biomarkers for humoral- as well as cellular immunity.

Tests used in leprosy diagnostics include the broadly investigated serological assay detecting IgM against PGL-I [13], [14]. Although this test is useful for detection of most multibacillary (MB) patients [15], [16], as the antibody levels correlate well with the bacillary load, detection of anti-PGL-I Ab has limited value in identifying paucibacillary (PB) leprosy patients [17]. In areas hyperendemic for leprosy more than 50% of young schoolchildren surveyed had positive anti-PGL-I responses [18]. Still, the vast majority of individuals with a positive antibody titer will never develop leprosy [13]. With respect to cellular responses in leprosy diagnosis, studies have focussed on *M. leprae*-unique antigens which can probe T-cell *M. leprae*-specific responses resulting in the identification of *M. leprae* (-unique) antigens that specifically induced IFN-γ production in *M. leprae* infected individuals [19], [20]. Combined with serology, the use of these IFN-γ release assays (IGRAs) provided significant added value since they identified the majority (71%) of PGL-I negative healthy household contacts in Brazil [21] while control individuals not exposed to *M. leprae* were IGRA-negative. Similar IGRAs allowed detection of the extent of *M. leprae* exposure along a proximity gradient in EC in one city in Brazil and in Ethiopia [22]–[24].

Although ELISA techniques, as used in IGRAs, are more widely applied than before, they still require laboratory facilities which are not available at all health centres in leprosy endemic areas. To accommodate ELISAs to field-applicable tests for leprosy diagnosis, we previously developed UCP-LFAs for detection of IFN-γ and IL-10 as well as antibodies against the *M. leprae*-specific phenolic glycolipid-I (PGL-I) for high-tech, laboratory-based microtiter-plate readers [25], [26]. Since IFN-γ, the hallmark cytokine of Th1 cells, has generally been assessed as a biomarker to detect anti-mycobacterial immunity, we first developed a IFN-γ-UCP-LFA [25]. Recently, IFN-γ induced protein 10 (IP-10) was found useful for detection of *M. tuberculosis* infection [27] and can also be used to indicate levels of *M. leprae* exposure and thereby the risk of infection and subsequent transmission [22], [23]. Furthermore, since IP-10 is produced in large quantities, facilitating the use of simplified test platforms such as LFA [28], we investigated its potential as an alternative to IFN-γ for leprosy diagnosis. Accordingly, we developed quantitative, dry reagent UCP-LFAs for field-detection of IP-10 and anti-PGL-I antibodies and evaluated these in a leprosy endemic area in Ethiopia.

## Materials and Methods

### Ethical statement

This study was performed according to ethical standards in the Helsinki Declaration of 1975, as revised in 1983. Ethical approval of the study protocol was obtained from the National Health Research Ethical Review committee, Ethiopia (NERC # RDHE/127-83/08) and The Netherlands (MEC-2012-589). Participants were informed about the study objectives, the required amount and kind of samples and their right to refuse to take part or withdraw from the study at any time without consequences for their treatment. Written informed consent was obtained from all study participants before venipuncture.

### Study participants

HIV-negative, newly diagnosed untreated leprosy patients and healthy endemic controls (EC) were recruited at the Armauer Hansen Research Institute (AHRI) in Addis Ababa, Ethiopia, The Leiden University Medical Center (LUMC) and the Erasmus Medical Center (EMC), The Netherlands from October 2011 until November 2012. Leprosy was diagnosed based on clinical, bacteriological and histological observations and classified by a skin biopsy evaluated according to the Ridley and Jopling classification [2] by qualified personnel. EC were assessed for the absence of signs and symptoms of tuberculosis and leprosy. Staff members working in the leprosy centers or TB clinics were excluded as EC. Mantoux-negative, healthy Dutch donors recruited at the Blood Bank Sanquin, Leiden, The Netherlands were used as nonendemic controls (NEC). None of these NEC had lived in or travelled to leprosy- or TB endemic areas, and, to their knowledge, had not experienced any prior contact with TB or leprosy patients.

### Recombinant proteins

*M. leprae* candidate genes were amplified by PCR from genomic *M. leprae* DNA and cloned using Gateway technology (Invitrogen, Carlsbad, CA) with pDEST17 expression vector containing an N-terminal histidine tag (Invitrogen) [29]. Purified recombinant proteins were produced as described [22], [29] and contained endotoxin levels below 50 IU per mg recombinant protein as tested using a Limulus Amebocyte Lysate (LAL) assay (Cambrex, East Rutherford, NJ). Recombinant proteins were tested to exclude protein non-specific T cell stimulation and cellular toxicity in IFN-γ release assays using PBMC of *in vitro* PPD-negative, healthy Dutch donors recruited at the Blood Bank Sanquin, Leiden, The Netherlands. None of these controls had experienced any known prior contact with leprosy or TB patients.

### Whole blood assays (WBA)

Within 3 hours of collection, venous heparinized blood (450 µl per well) was incubated in 48-well plates at 37°C at 5% CO_2_, 90% relative humidity with 50 µl of antigen solution (100 µg/ml). After incubation periods of 1 h, 4 h, 6 h or 24 h (as indicated), 150 µl of supernatants were removed from each well and frozen in aliquots at −20°C until further analysis.

### Synthetic PGL-I and *M. leprae* whole cell sonicate (WCS)

Synthetic PGL-I (ND-O-HSA) and *M. leprae* whole cell sonicate were generated with support from the NIH/NIAID Leprosy Contract N01-AI-25469 (available through the Biodefense and Emerging Infections Research Resources Repository listed at http://www.beiresources.org/TBVTRMResearchMaterials/tabid/1431/Default.aspx). Disaccharide epitope (3,6-di-O-methyl-β-D-glucopyranosyl(1→4)2,3-di-O-methylrhamnopyranoside) of *M. leprae* specific native PGL-I glycolipid was synthesized and coupled to human serum albumin (ND-O-HSA) as previously described by Cho *et al.* [30]. Inactivated (irradiated) armadillo-derived *M. leprae* whole cells were probe sonicated with a Sanyo sonicator to >95% breakage.

### PGL-I ELISA

IgM antibodies against *M. leprae* PGL-I were detected with natural disaccharide of PGL-I linked to human serum albumin (ND-O-HSA (500 ng/well in 50 µl) provided through the NIH/NIAID Leprosy Contract N01-AI-25469) as previously described [31]. Serum dilutions (50 µl/well; 1∶800) were incubated at RT for 120 min in flat-bottomed microtiter plates (Nunc) coated with NDO-HSA. After washing diluted enzyme linked secondary antibody solution (anti-human IgG/IgM/IgA – HRP; Dako, Heverlee, Belgium; 50 µl/well) was added to all wells and incubated at RT for 120 min. After washing diluted TMB solution (50 µl/well) was added to all wells and incubated in the dark for 15 min at RT. The reaction was stopped by adding 50 µl/well 0.5 N H_2_SO_4_. Absorbance was determined at wavelength of 450 nm. Samples with a net optical density at 450 nm (OD) above 0.149 were considered positive. The ELISA performance was monitored using a positive and negative control serum samples on each plate.

### Cytokine ELISAs

For ELISAs 96 well Nunc MaxiSorp microtitre-plates were used and the presence of biotinylated antibody was detected enzymatically using streptavidin-HRP (horse-radish peroxidase): **IFN-γ** was determined using anti-IFN-γ coating Ab mAb mO-13-32-22 (U-CyTech Biosciences, Utrecht, the Netherlands) and biotinylated anti-IFN-γ pAb pB-15-43-13 (U-CyTech Biosciences) as detection Ab. Culture supernatants were diluted 1∶2 in buffer (1% BSA/PBS) and the cut-off value to define positive responses was set beforehand at 100 pg/ml. The assay sensitivity level was 40 pg/ml. Values for unstimulated cell cultures were typically <20 pg/ml. **IP-10** was determined using anti-IP-10 capture Ab (clone B-C50) and biotinylated anti-IP-10 detection Ab (clone B-C55; Diaclone, France) in culture supernatants diluted 1∶100 with dilution buffer. The cut-off value to define positive responses was set beforehand at 2,000 pg/ml. The assay sensitivity level was 40 pg/ml. Values for unstimulated cell cultures of NEC were typically <2,000 pg/ml. **IL-10** was determined using anti-IL-10 mAb mO-13-10-12 (U-CyTech Biosciences) as coating Ab and biotinylated anti-IL-10 pAb mB-15-10-26 (U-CyTech Biosciences) as detection Ab in culture supernatants diluted 1∶2. The cut-off value to define positive responses was set beforehand at 100 pg/ml. The assay sensitivity level was 10 pg/ml. Concentration values for unstimulated whole blood were typically ≤10 pg/ml.

### Upconverting phosphor (UCP) conjugates and LF strips

UCP conjugates specific for cytokines IP-10, IL-10, IFN-γ were prepared following earlier described protocols [26], by conjugating 5 µg anti-IP-10 (BC-50; Diaclone), 20 µg anti-IL-10 mAb (coating mAb in ELISA, mO-13-10-12; U-CyTech) or 25 µg anti-IFN-γ (BB-1; Diaclone) per 1 mg carboxylated UCP particles, respectively. Wet UCP conjugates were stored at a concentration of 1 mg/ml at 4°C. An UCP-IP-10 dry conjugate was made by drying 100 ng in a 5% sucrose matrix overnight at 37°C in 0.65 ml U-shape polypropylene tubes (Ratiolab tubes for 96-well micro test plate, VWR International, Amsterdam, The Netherlands); dried materials were stored in aluminum foil bags (Lamigrip pouches Overtoom International, Den Dolder, The Netherlands) with silica dry pellets at ambient temperature [6], [32]. Reporter conjugates for detection of humoral immune response, an IgM- and Ig-specific UCP conjugates, were prepared as described earlier [9], [26] by conjugation of 25 µg goat anti-human IgM (I0759; Sigma-Aldrich, Saint Louis, MO, USA), protein-A (Repligen Corp.) or IgG/IgM/IgA/Kappa/Lambda–HRP (Dako), respectively. Wet conjugates were stored at a concentration of 1 mg/mL at 4°C. Freeze dried pellets, so-called lyospheres, containing 100 ng UCP^protein A^ conjugate were produced (Biolyph LLC, Hopkins, MN, USA) and stored in vacuum-sealed glass vials as described earlier [33]. LF strips (4 mm width) for IP-10, IL-10 and IFN-γ were prepared with a test (T) line at 2.0 cm comprised of 50 ng anti-IP-10 BC-55 (Diaclone), 700 ng anti-IL-10 mAb mO-10-10-28 (U-CyTech Biosciences) or 200 ng anti-IFN-γ BG-1 (Diaclone) respectively. The antibody pairs were identical to those used for ELISA but not containing a biotin hapten. LF strips for cytokine detection were further provided with a goat anti-mouse pAb (M8642; Sigma-Aldrich) flow-control (FC) line of respectively 100 ng and 200 ng at 2.5 cm. LF strips for detection of antibodies against PGL-I were provided with 50 ng synthetic PGL-I (ND-O-HAS) on the test (T) line and 100 ng rabbit anti-goat IgG (G4018; Sigma-Aldrich) on the flow-control (FC) line. LF strips for IP-10 and PGL-I multiplex detection were prepared using the same compositions as the strips for the individual targets, but now were provided with two T- and two FC-capture lines. Capture lines were separated by 4 mm located at 1.5 (T1, IP-10), 1.9 (T2, PGL-I), 2.7 (FC1, goat anti-mouse), and 2.3 cm (FC2, rabbit anti-goat).

### UCP-LFA for cytokine detection

The UCP-LFAs for cytokine detection (IFN-γ, IL-10, IP-10) comprise two phases, designated solution phase and immunochromatography phase [26]. Solution phase: 10 µl of 100-fold diluted sample (translating to 0.1 µl undiluted sample) for IP-10 and 10 µl undiluted sample for IL-10 and IFN-γ is mixed with 90 µl High Salt Lateral Flow (HSLF) buffer (100 mM Hepes pH 7.2, 270 mM NaCl, 1% BSA (w/v), 0.5% Tween-20 (v/v)) containing 100 ng specific UCP reporter conjugate and incubated for 60 min on a thermoshaker at 37°C and 900 rpm. The immunochromatography phase: the above mixture is applied to cytokine specific LF strip and allowed to flow for at least 30 min. After immunochromatography, LF strips are scanned in a Packard FluoroCount microtiterplate reader adapted with an infrared laser. Upon IR excitation (980 nm), UCP reporter particles emit green light detectable using a 550 nm band pass filter. Results are displayed in histograms in relative fluorescence units (RFUs) measured at Test and Flow-Control lines, or as the ratio value between Test (T) and Flow-Control (FC) RFUs using Lateral Flow Studio software V 3.3.5 (QIAGEN Lake Constance GmbH). For strip analysis in Ethiopia a lightweight portable LF strip reader with UCP capability was used (UCP-Quant, an ESEQuant *LFR* reader custom adapted with IR diode; QIAGEN Lake Constance GmbH, Stockach, Germany) [6]. Best reproducibility is obtained when analyzing completely dry LF strips, whereas wet LF strips generate lower T and FC signals. Ratio values between wet- and dry-scanned strips are not significantly different when scanned with readers with sufficient sensitivity that contain a high power IR laser and an adjustable photo multiplier [34]. Since wet-format assays require a sonication step, not suitable for field applications [6], the IP-10-UCP-LFA was adapted to allow implementation of dry reagents (dry conjugate and lyophilized buffer) similar as described for Schistosomiasis [6] and RSV [33]. Next, the dry-format IP-10-UCP-LFA was transported to Ethiopia at ambient temperature and used by local staff after short instruction. In order to evaluate the field performance of these dry-format UCP-LFAs at the Ethiopian site, a lightweight dedicated UCP-LF strip analyzer was provided.

### UCP-LFA for anti-PGL-I antibody detection

For detection of anti-PGL-I IgM antibodies two protocols were used: a rapid sequential flow protocol without incubation using the UCP^protein-A^ or UCP^αIgG/IgM/IgA/Kappa/Lambda^ conjugate, or a two phase protocol similar to the above described protocol for cytokine detection only using UCP^αIgM^ instead of cytokine-specific UCP conjugates. The sequential flow protocol using the UCP^protein-A^ conjugate is referred to as consecutive flow (CF) as described [8], [9], [35]. The CF protocol comprised three sequential flow steps: first 40 µl of a diluted clinical sample (2.5% (v/v) in HSLF assay buffer), after 2 min followed by a wash step with 20 µl HSLF and a final flow after 5 min with 70 µl UCP-conjugate (100 ng in HSLF). Multiple strips can be handled simultaneously by prefilling 96 well ELISA microtitre-plates (Nunc MaxiSorp) with the appropriate three solutions and transferring LF strips from one well to the other. Immunochromatography is allowed to continue for at least 30 min before LF strips are analyzed (see above). For the dry-format UCP-LFA to detect anti-PGL-I antibodies, dry UCP^prot-A^ reagent in the form of lyospheres [2] was used.

### UCP-LFA for simultaneous (multiplex) cytokine and antibody

Simultaneous detection of IP-10 and anti-PGL-I IgM was performed following the two phase protocol described above for cytokine detection. The solution phase comprised the incubation (60 min; 37°C; 900 rpm) of 10 µL 100-fold diluted sample (translating to 0.1 µL of the original undiluted clinical sample) with 90 µl HSLF buffer containing 100 ng of the UCP^αIP-10^conjugate (wet) and 100 ng of the UCP^αIgM^ conjugate. The immunochromatography phase was identical to that described for the cytokine-only testing protocol and allowed to continue for at least 30 min before analysis of LF strips (see above). Note that the above protocol may not be applicable when performing antibody detection with the UCP^protein-A^ conjugate due to unwanted interaction of protein-A with the UCP^αIP-10^ conjugate [26].

### Statistical analysis

Differences in cytokine concentrations between test groups were analysed with the two-tailed Mann-Whitney U test for non-parametric distribution using GraphPad Prism version 5.01 for Windows (GraphPad Software, San Diego California USA;www.graphpad.com). For correlations *R^2^* was calculated with the Pearson correlation using GraphPad Prism version 5.01. The statistical significance level used was p≤0.05.

## Results

### Combined cytokine profiles in response to *M. leprae* antigens

*M. leprae* unique antigens can be used to indicate *M. leprae* exposure using IFN-γ and IP-10 as read-outs [22], [23], [36]. Also, IFN-γ and IP-10 are associated with Th1-mediated protection against mycobacteria, whereas the anti-inflammatory cytokine IL-10 dampens Th1 cells' responses [37]–[39]. In view of the high levels of IP-10 produced compared to IFN-γ [22], [28] and since, in contrast to IFN-γ, IP-10 is not affected by low CD4 counts in TB patients with HIV [28], we investigated whether IP-10, as an alternative to IFN-γ, can be applied as a pro-inflammatory biomarker.

**Figure 1:**
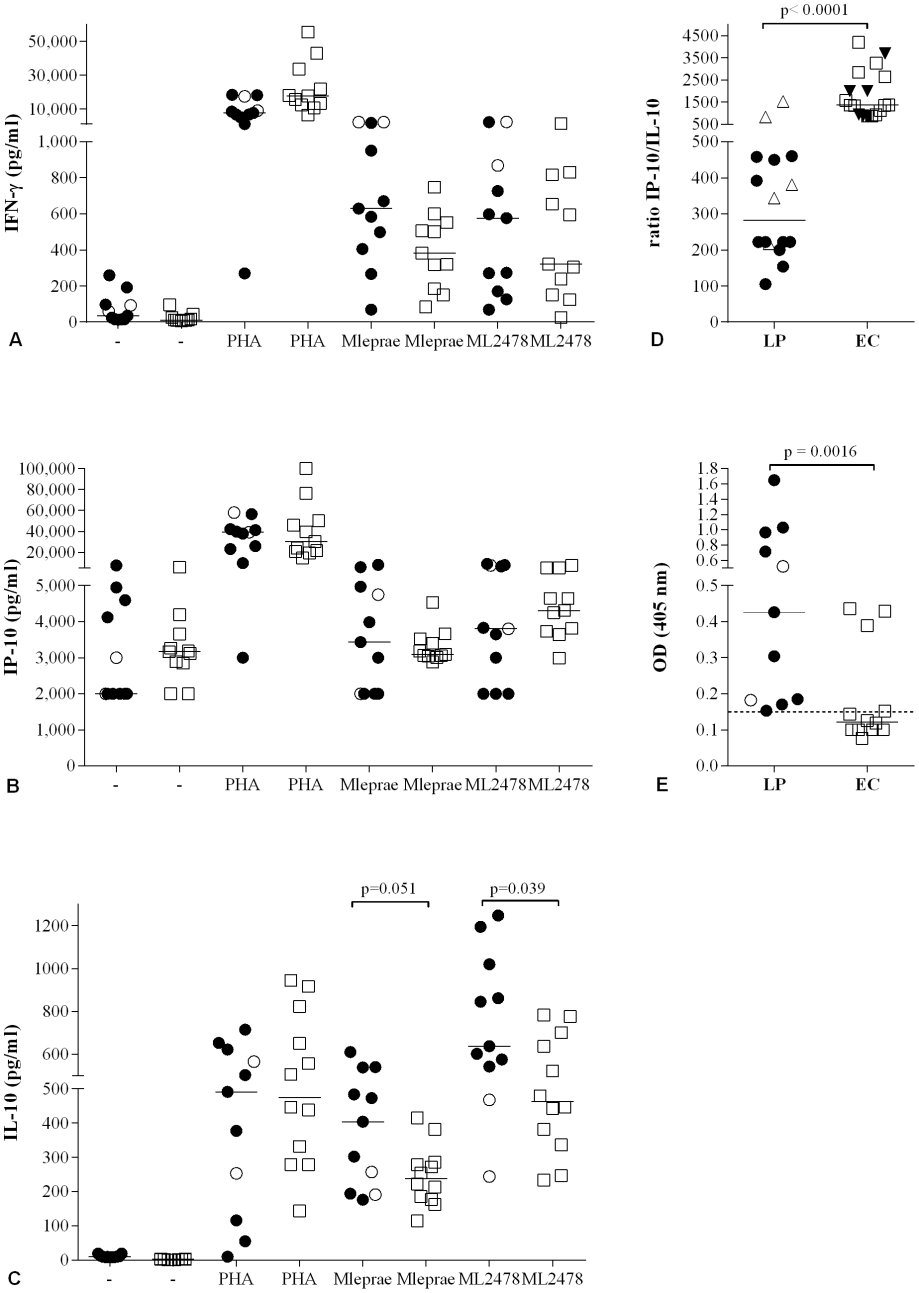
Combined cytokine profiles in response to *M. leprae*. Production of IFN-γ (**A**), IP-10 (**B**) and IL-10 (**C**) determined by ELISA, in response to medium (-), PHA, *M. leprae* WCS or the *M. leprae*-unique protein ML2478 in 24 h WBA for Ethiopian leprosy patients (n = 11: 2 BT (○) and 9 BL (•), and healthy endemic controls (EC; n = 12; □). For comparison between BT and BL, significant differences were found for *M. leprae* WCS (Mlep) induced IFN-γ responses (p = 0.036) and ML2478 induced IL-10 responses (p = 0.035). (**D**): IP-10/IL-10 ratios are depicted for unstimulated samples after 24 h {LP (•) and EC (□)} or after 1 h WBA {LP (▵) and EC (▾)}. (**E**): Anti-PGL-I antibodies for BL (○) and BT (•) patients were detected by ELISA using natural disaccharide of PGL-I linked to HSA [31] (ND-O-HSA). Optical density (OD_450_) readings were performed using 1∶800 serum dilutions. Median values per group are indicated by horizontal lines. The cut-off for positivity is indicated by the dashed horizontal line.

To evaluate the combined diagnostic value of IL-10, IP-10 and IFN-γ, we first determined their concentrations by ELISAs in 24 h WBA of 11 Ethiopian leprosy patients (9 BL, 2 BT) and 12 EC. In addition, anti-PGL-I antibodies were determined for each individual as well (Figure 1). The IP-10 production measured in WBA displayed a pattern similar to that of IFN-γ, although the overall IP-10 concentrations were much higher: median levels of both cytokines in response to *M. leprae* and ML2478 in patients' WBA were not significantly different from those for EC in this leprosy endemic area. These data are consistent with our previous findings, leading to the use of IFN-γ/IP-10 production in response to ML2478 to determine the level of exposure to *M. leprae* irrespective of infection [22].

In contrast, IL-10 concentrations in response to ML2478, were significantly lower for EC (Figure 1C). Since the balance of pro- and anti-inflammatory cytokines in response to *M. leprae* regulates the clinical outcome after infection, diagnostic tests for leprosy measuring both type of responses will be helpful in the decision on which individuals need (preventive) treatment. IP-10/IL-10 ratios for stimulated and unstimulated WBA samples demonstrated significantly different values between patients and EC, in particular for unstimulated samples (Figure 1D). Finally, detection of a biomarker for humoral immunity, anti-PGL-I antibody levels, demonstrated significantly higher titers for leprosy patients, further contributing to a discriminating profile between leprosy patients and EC in leprosy endemic areas (Figure 1E).

### Kinetics of cytokine production in WBA

**Figure 2:**
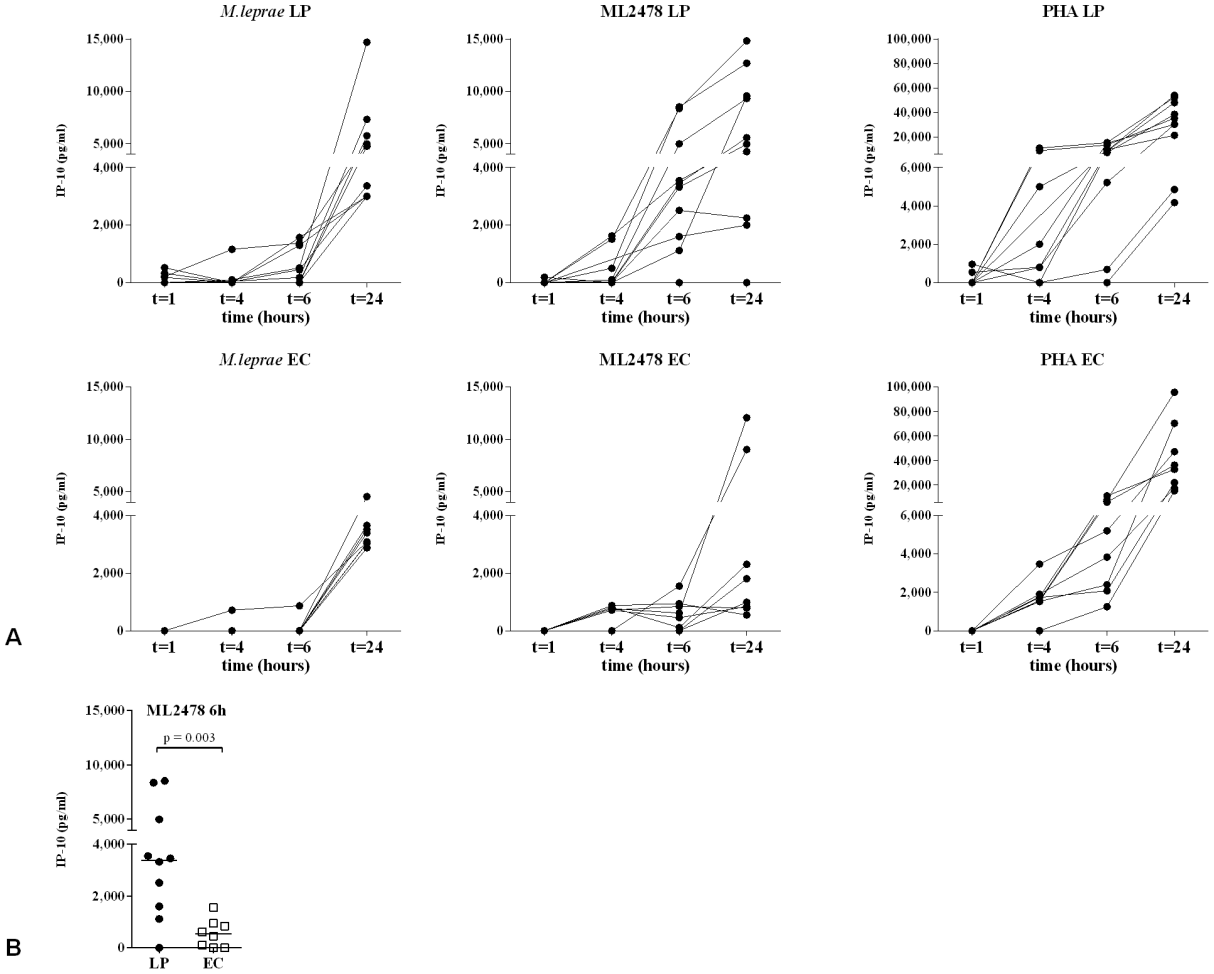
Kinetics of IP-10 production in WBA. (**A**): IP-10 concentrations produced in stimulated whole blood cultures of leprosy patients (upper panel; LP; n = 10: 5 BL (Ethiopia); 2 BT (Ethiopia); 3 BT (The Netherlands) and healthy endemic controls (lower panel; EC, n = 8) in response to *M. leprae* WCS (left panel; 10 µg/ml), *M. leprae* unique protein ML2478 (middle panel; 10 µg/ml) and PHA (right panel; 1 µg/ml). IP-10 concentrations were determined by ELISA after 1 h, 4 h, 6 h and 24 h antigen stimulation. Values on the y-axis are concentrations corrected for background values. (**B**): Comparison of IP-10 concentrations determined by ELISA after 6 h stimulation with ML2478 (10 µg/ml) of whole blood samples.

Since short overall test-to-result times are preferred for diagnostic assays, the supernatants of WBA of Ethiopian leprosy patients and EC were analyzed for the presence of IFN-γ, IL-10 and IP-10 after 1 h, 4 h, 6 h and 24 h stimulation. For IFN-γ and IL-10, levels that varied significantly from unstimulated samples were only detected after 24 h (data not shown). For IP-10, however, already after 6 h significant production was observed in antigen stimulated samples (Figure 2). Important to note is that after 6 h, IP-10 levels in ML2478-stimulated samples were significantly higher (p = 0.02) in patients compared to EC (Figure 2B), whereas no distinctive responses were observed for IFN-γ at that time point. PHA-induced IP-10 levels were high for all individuals after 6 h and substantial IP-10 levels were only detectable in *M. leprae*-stimulated samples after 24 h. Thus, besides the higher levels of IP-10, also the shorter whole blood assay time required render IP-10 combined with ML2478 or as ratio with IL-10 directly in serum, a preferred pro-inflammatory biomarker to discriminate between leprosy patients and EC.

### Development and evaluation of UCP-LFAs

**Figure 3:**
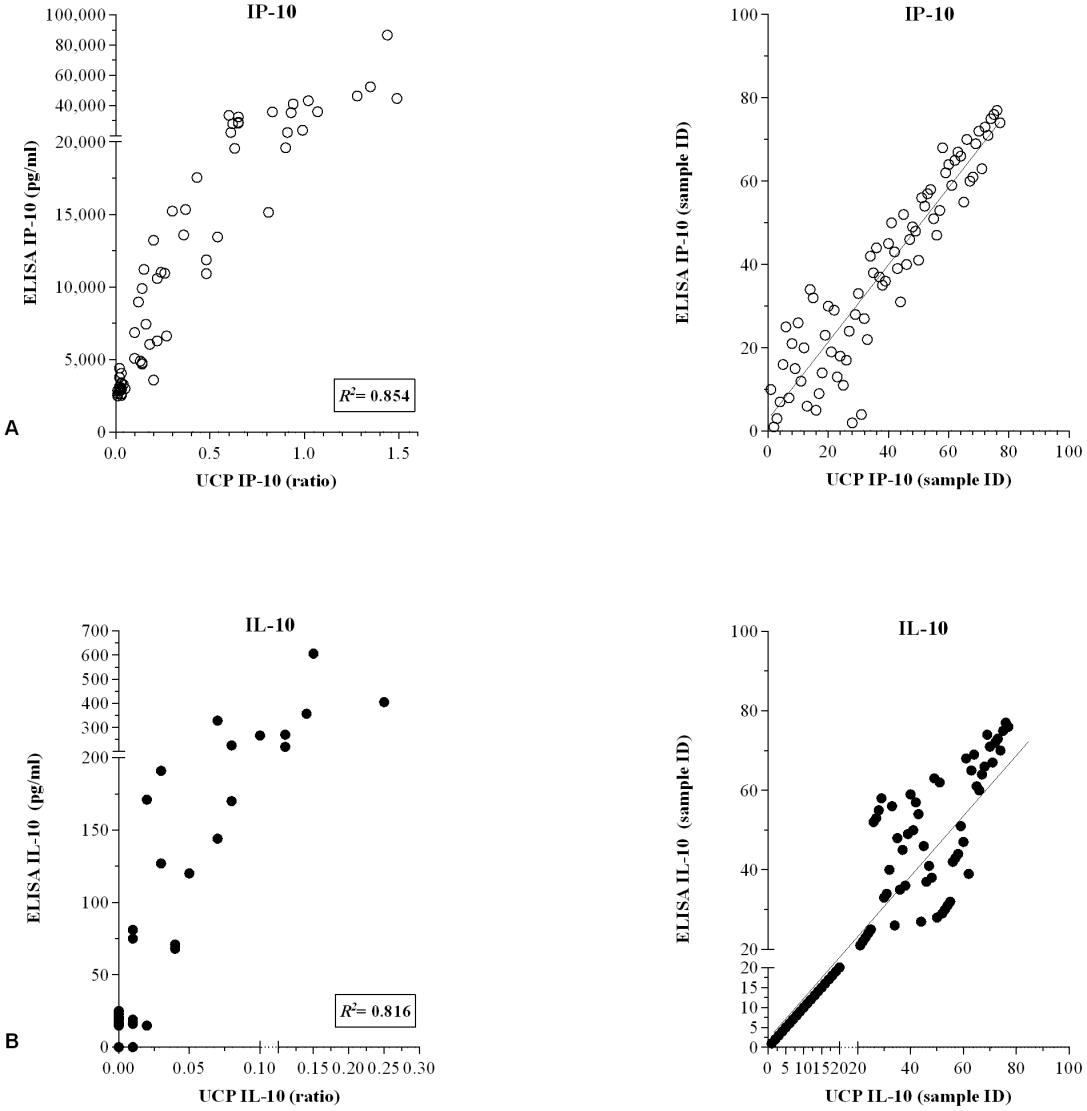
Correlation between ELISAs and UCP-LFAs. Levels of IP-10 (**A**) and IL-10 (**B**) in 24 h whole blood samples of 77 *M. leprae* (antigen), LPS and PHA stimulated WBA samples of Dutch healthy controls were simultaneously determined by ELISAs and wet-format UCP-LFAs. **Left panels**: results for ELISAs are indicated in pg/ml (ELISA) or as the ratio of the relative fluorescence units (RFUs) measured at Test and Flow-Control lines (UCP-LFA). *R^2^* equals the square of the Pearson correlation coefficient. **Right panels**: Spearman ranking.

For detection of IFN-γ, IL-10 as well as antibodies against *M. leprae* PGL-I, we previously developed up-converting phosphor lateral flow assays (UCP-LFAs) [25], [26]. Because of the potential of IP-10 to identify *M. leprae* infection in a shorter test-to-result time as well as the value of IP-10/IL-10 ratios, we now selected IP-10 for UCP-LFA development, using the wet-format for IL-10 described previously [26]. Validation of these IL-10 and IP-10 UCP-LFA by comparison to ELISAs utilizing the same antibody pairs and antigen-stimulated WBA samples of non-endemic controls (NEC), demonstrated good correlations between UCP-LFAs and ELISAs for IP-10 and IL-10 (*R^2^* 0,854 and *R^2^* 0,816, respectively; Figure 3).

**Figure 4:**
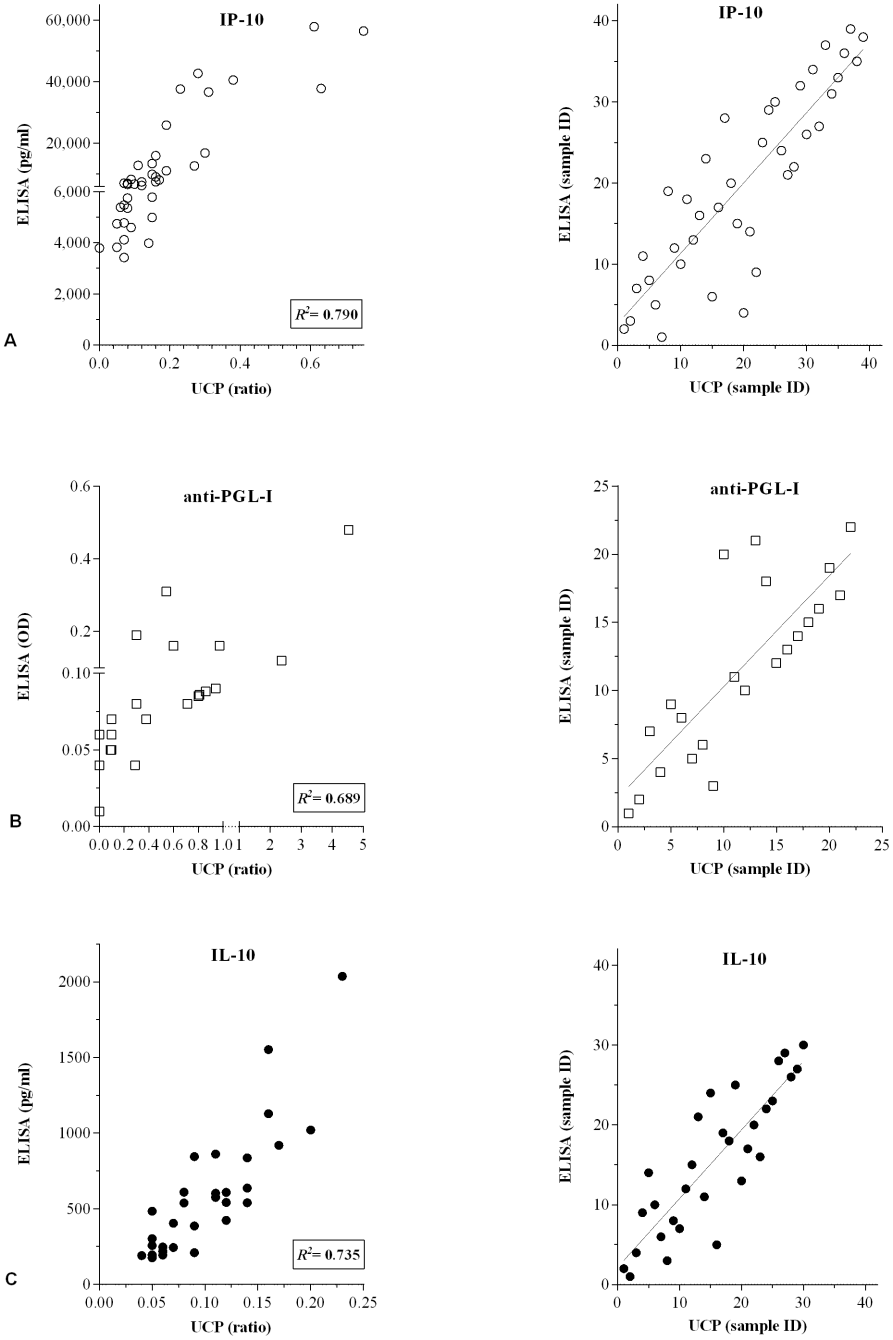
Correlation between ELISAs and UCP-LFAs. Levels of IP-10 (**A**; n = 40), anti-PGL-I antibodies (**B**; n = 22) or IL-10 (**C**; n = 40) in WBA samples were simultaneously determined by ELISAs and UCP-LFAs in Ethiopia using dry-format (**A**, **B**) or wet format (**C**) UCP-LFAs. For cytokine analysis (**A** and **C**), samples of Ethiopian leprosy patients (2 BT and 8 BL) that were unstimulated or stimulated with *M. leprae* WCS, ML2478 or PHA were used. For anti-PGL-I antibodies (**B**), samples of Ethiopian leprosy patients (2 BT and 8 BL) and healthy endemic controls (n = 12) were used. **Left panels**: results for ELISA are indicated in pg/ml (**A**, **C**) or OD_450_ (**B**) or as the ratio of the relative fluorescence units (RFUs) measured at Test and Flow-Control lines (UCP-LFA). *R^2^* equals the square of the Pearson correlation coefficient. Correlation was calculated for samples with ELISA values higher than the cut-off threshold. **Right panels**: Spearman ranking.

In view of the greater stability in the field, dry assay format IP-10-UCP-LFA were produced and evaluated in Ethiopia as well: IP-10 values obtained in both wet and dry assays showed a good correlation (*R^2^* 0,790; Figure 4A) indicating the value for field application of the dry-format IP-10-UCP-LFA. Similarly, the unstimulated WBA samples were locally (in Ethiopia) tested for the presence of antibodies against PGL-I as well. Quantitive analysis of the UCP^prot-A^ ratios and ELISA OD values correlated well (*R^2^* 0.689; Figure 4B) indicating 100% agreement in respect to serological status of the samples (qualitative analysis).

To further evaluate UCP-LF applications with this Ethiopian sample set, IL-10 levels of 84 samples (21 patients, 3 stimuli and medium) were also tested, using the available wet-format IL-10-UCP-LFA in parallel with ELISA. Since the IL-10-UCP-LFA was used with 100-fold larger sample input than the IP-10 assay, some of the discrepancies observed for IL-10 between ELISA and UCP-LF assay were probably due to particulate material present in WBA samples. Despite these differences, IL-10-UCP-LFA and ELISA correlated well (*R^2^* 0,735; Figure 4C).

**Figure 5:**
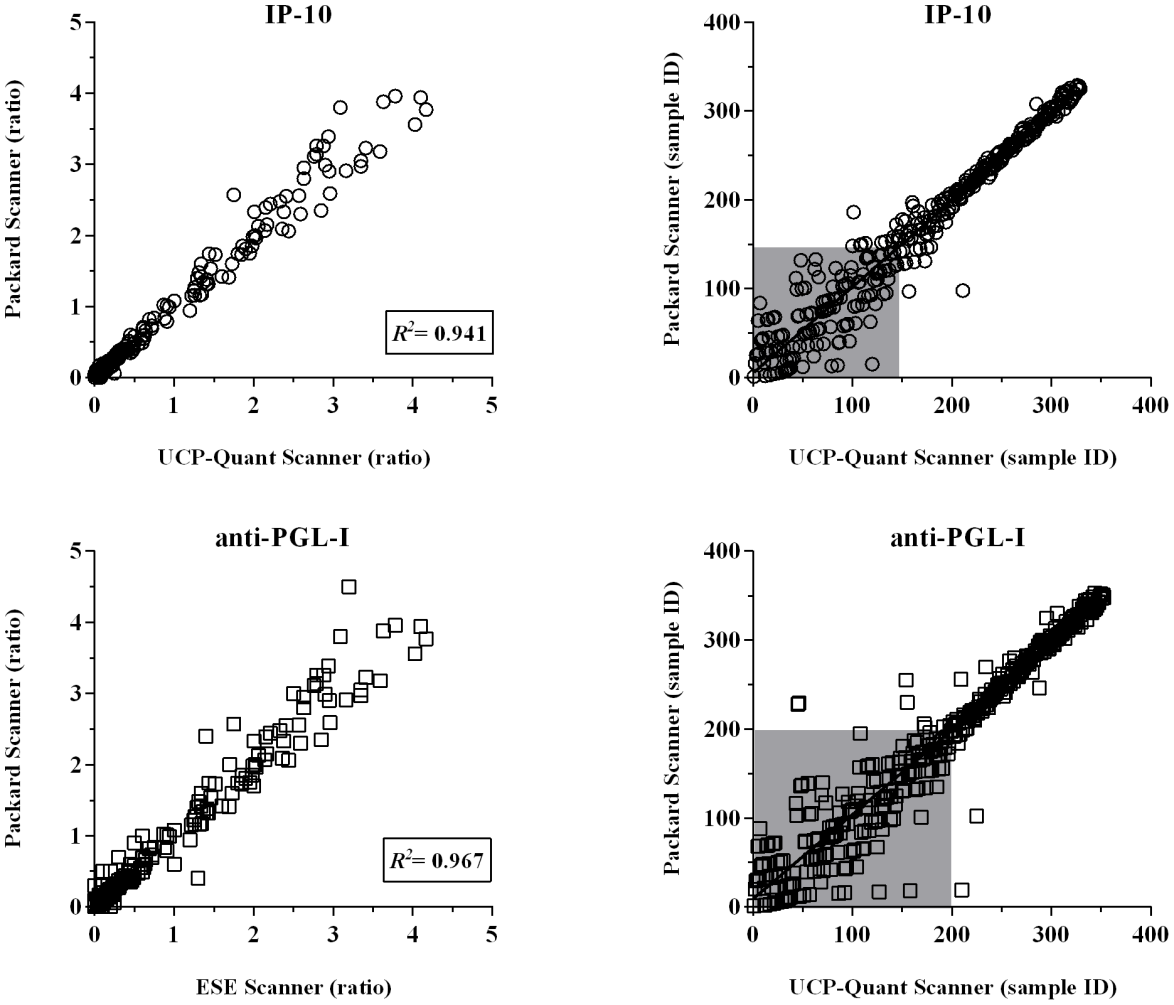
Performance of the portable lightweight UCP-Quant LF strip reader. Dry-format UCP-LFAs were performed for single detection of IP-10 and anti-PGL-I antibodies in an Ethiopian field setting (Figure 3). LF strips were analyzed using a portable reader (UCP-Quant). Subsequently, LF strips were shipped to The Netherlands and re-analysed using a dedicated lab-based FluoroCount microtiterplate reader (Packard) adapted for reading UCP-LF strips. **Left panel**: results are indicated as the ratio of the relative fluorescence units (RFUs) measured at Test and Flow-Control lines. *R^2^* equals the square the Pearson correlation coefficient. **Right panel**: Spearman ranking. The grey box indicates samples scoring values below the specificity threshold.

For direct comparison of single UCP-LFAs performance in a field- versus laboratory setting, the UCP-LF strips for IP-10 and anti-PGL-I antibodies analyzed in Ethiopia were sent to The Netherlands and re-analysed using a dedicated, high-tech UCP scanner, a Packard FluoroCount microtiter-plate reader adapted with an infrared laser (980 nm) capable to scan 20 strips simultaneously. Comparison of ratios obtained in both tests showed an excellent correlations between both scanners (IP-10: *R^2^* 0,960 and PGL-I: *R^2^* 0,901; Figure 5), demonstrating that the UCP-LF strips can be stored as permanent record allowing re-analysis in a reference laboratory. Since leprosy endemic areas are often short of sophisticated laboratories, these results indicate that UCP-LFAs represent robust test suitable for resource-poor settings.

### Multiplex UCP-LFA for detection of IP-10 and anti-PGL-I antibodies

**Figure 6:**
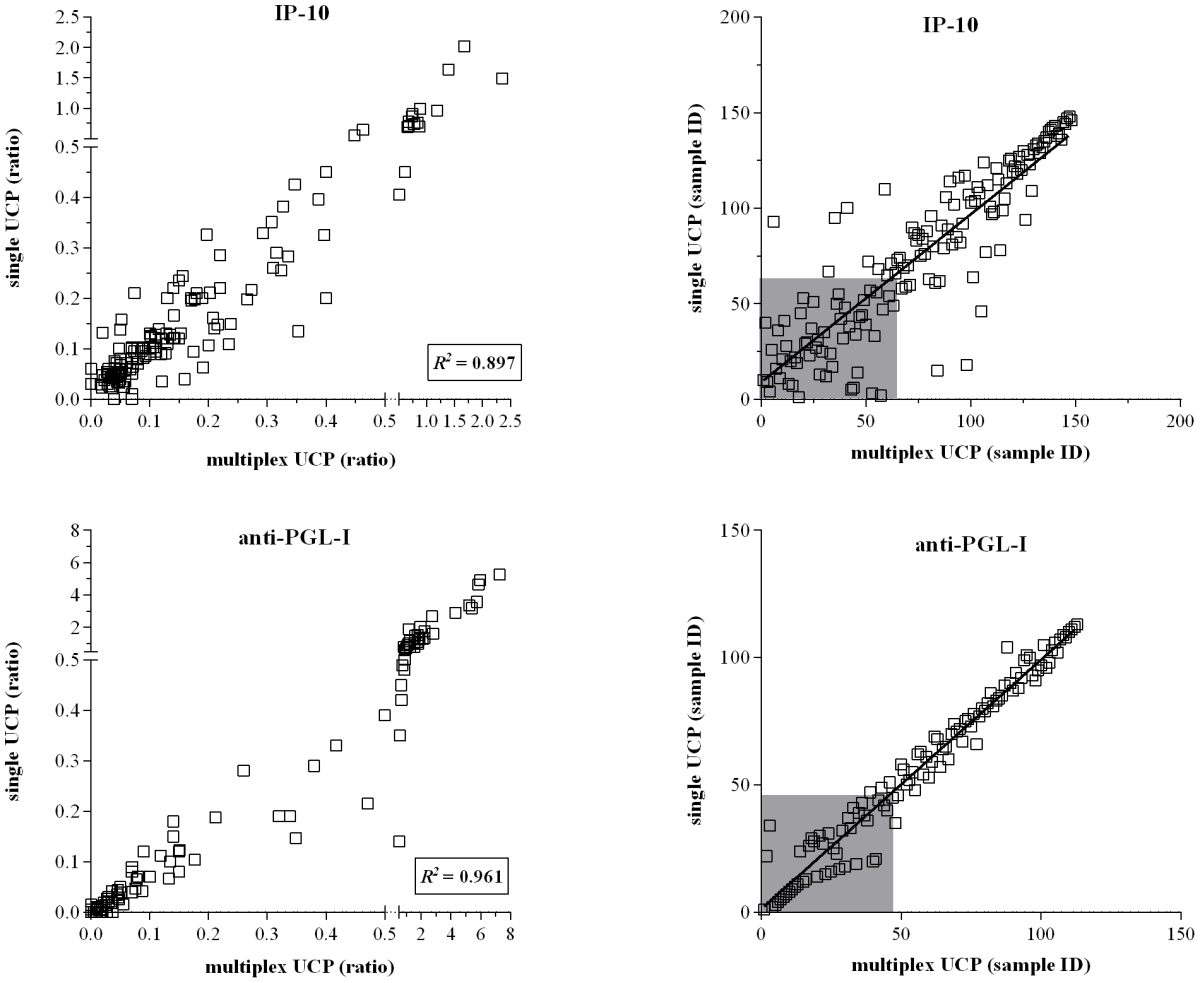
Comparison between single and multiplex UCP-LFAs. UCP-LFAs were performed for single or multiplex detection of IP-10 (upper panel; n = 149 samples) and anti-PGL-I (lower panel; n = 115 samples) using *M. leprae* antigen-stimulated WBA samples of Dutch and Ethiopian leprosy patients. Simultaneous detection of IP-10 and anti-PGL-I IgM was performed following the two phase protocol using the UCP^αIP-10^conjugate and the UCP^αIgM^ conjugate. **Left panel**: Results for UCP-LFAs are displayed as the ratio of the relative fluorescence units (RFUs) measured at Test and Flow-Control lines. *R^2^* equals the square of the Pearson correlation coefficient. **Right panel**: Spearman ranking. The grey box indicates samples scoring values below the specificity threshold.

IP-10 levels as well as anti-PGL-I antibody concentrations were present in high concentrations allowing reliable detection even with small amounts of serum thereby improving the robustness in field assays. To further simplify the use of the UCP-LFA for leprosy diagnostics in a field setting, we next developed a multiplex UCP-LFA for simultaneous detection of anti-PGL-I antibodies and IP-10 in whole blood samples, analogous to the earlier described anti-PGL-I/IL-10 multiplex UCP-LFA [26]. The advantage of this specific chemokine/antibody combination is that similarly diluted serum samples can be used, facilitating multiplex analysis of cellular and humoral immunity. For extensive comparison of single and multiplex UCP-LFAs Dutch leprosy patients' WBA samples were used as well to accommodate for more samples. Multiplex UCP-LFA and the single UCP-LFA for IP-10 and anti-PGL-I antibodies showed good correlations (*R^2^* 0,961 and 0, 897; Figure 6) demonstrating the applicability of this multiplex UCP-LFA.

## Discussion

Effective diagnostics are essential tools for the control, elimination and eradication of neglected diseases such as leprosy. Since leprosy endemic areas are often short of sophisticated laboratories, it is imperative to develop diagnostic tests for early detection of *M. leprae* infection that are suitable for field settings. The main requisite for such diagnostic tests is the selection of suitable biomarkers. WBA using *M. leprae*(-specific) antigens induce a ‘fingerprint’ of (the ratio of) pro- and anti-inflammatory cytokines that, combined with detection of anti-PGL-I antibodies, can be used as a biomarker profile for *M. leprae* infection.

Notwithstanding the frequent use of IFN-γ, IP-10 represents an equally valid biomarker for pro-inflammatory responses to mycobacteria [22], [23], [27], [36], [40], [41]. This chemokine is produced by various cell types, including monocytes/macrophages, and is involved in recruitment of lymphocytes and neutrophils to sites of inflammation. IP-10 can be used to differentiate between high and low *M. leprae* exposure levels [22] and it also provides a biomarker associated with type 1 reactions (T1R) in leprosy patients [42], [43]. Moreover, IP-10, is much less influenced by CD4 cell count and, in contrast to IFN-γ, can be used in HIV^+^ individuals [28]. Considering the similarities in IP-10 responses of *M. leprae*- and *M. tuberculosis* infected individuals, and the high concentrations in which it is produced, we developed a UCP-LFA for IP-10 and investigated its diagnostic potential for leprosy (this study) and TB in Africa (Corstjens *et al.*, in preparation). Although most IGRAs require an antigen stimulation time of at least 24 h, we here demonstrate that IP-10, in contrast to IFN-γ, already showed a significant divergence between Ethiopian leprosy patients and EC after 6 h stimulation with the *M. leprae*-unique protein ML2478. This considerably reduces the overall assay time and could conveniently provide a sample-to-result on the same day.

Since host immunity and immuno-pathogenicity in response to *M. leprae* comprises multifaceted interactions between a diversity of cells secreting different molecules, it is rather unlikely that only a single compound is linearly correlated to protection or to disease progression [44]. Diagnostic tests that determine ratios of different types of cytokines will therefore be informative regarding disease development after *M. leprae* infection [19], [45] as was previously illustrated by IFN-γ/IL-10 and IFN-γ/IL-17 ratios in *Mtb* infected individuals [46], [47], but also for the development of T1R [42]. Relatedly, another valuable observation made here was the significant difference in IP-10/IL-10 ratios in sera of leprosy patients and EC, even without antigen stimulation. These data illustrate that the proportion of pro- to anti-inflammatory cytokines is consistent with clinical outcome after infection. Consequently, over time changes in the IP-10/IL-10 ratio for one individual will provide relevant clinical information with respect to the outcome of infection.

Dry-format UCP-LFAs are ideally suited for performance in the field and can be shipped and stored conveniently at ambient temperature and have prolonged shelf life of more than two years in African settings [6]. In this study we selected IP-10 and anti-PGL-I antibodies for field-evaluation of the dry-format UCP-LFA, and development of dry-format UCP-LFA for more analytes is in progress. This evaluation showed that both dry-format UCP-LFAs were equally sensitive as ELISAs and could be applied in the concentration range of 100 to >100,000 pg/ml. Also, the availability of affordable and portable UCP-LF strip readers showed suitability of the assay in field settings where ELISA equipment is not available or is more challenging to use. The LF strips were read with an easy to operate, portable reader that allows full instrument-assisted assay analyses avoiding operator bias. Due to the chemical stability of the assay components, the strips can be kept in patients' files and read again after long periods of time.

Besides the speed and ease of performance, another advantage of the UCP-LFA is that multiple analytes can be detected on the same LF-strip. Feasibility of multiplexed analysis was demonstrated previously for IL-10 and anti-PGL-I antibodies in spiked sera [26]. In this study multiplexing was successfully shown for IP-10 and anti-PGL-I antibodies in whole blood samples. Although the current UCP-LFA conditions for IL-10 quantitation demand a 100-fold larger sample input than the IP-10 assay, a single strip allowing quantitative detection of IP-10, IL-10 as well as anti-PGL-I antibody detection is feasible. Revision of the position (distance from the sample pad) and antibody load of the test lines, would allow the use of 1 µL samples instead of the currently applied 0.1 and 10 µL for IP-10 and IL-10 respectively. Moreover, multiplexing can be achieved by running two or more LF strips from a single sample in parallel as was for instance described for a simple multiple channel device running ten UCP-LF strips from a single sample [11].

This study describes the first steps towards development of a UCP-LFA as a field test measuring pro- and anti-inflammatory cellular- as well as humoral immunity to *M. leprae*, thereby including read-outs for multiple classifications of the leprosy spectrum. Such tests can be useful tools in leprosy control programs for classification of leprosy and allow early diagnosis of leprosy or leprosy reactions, leading to timely treatment and reduced transmission.
